# Effect of Fly Ash on Strength and Stiffness Characteristics of Controlled Low-Strength Material in Shear Wave Monitoring

**DOI:** 10.3390/ma14113022

**Published:** 2021-06-02

**Authors:** Sang-Chul Kim, Dong-Ju Kim, Yong-Hoon Byun

**Affiliations:** 1 School of Agricultural Civil & Bio-Industrial Engineering, Kyungpook National University, Daegu 41566, Korea; kimsc@knu.ac.kr; 2 School of Civil, Environmental and Architectural Engineering, Korea University (Formerly Kyungpook National University), Seoul 02841, Korea; kyrix1028@korea.ac.kr

**Keywords:** CLSM, compressive strength, elastic modulus, fly ash, shear wave

## Abstract

Fly ash, the main component for controlled low-strength material (CLSM), has physical and chemical characteristics according to the resources used in the thermal power plant, and thus fly ash type can influence the physical and strength properties of CLSM. This study investigates the effect of fly ash type on the engineering properties of CLSM and establishes relationships between mechanical properties of CLSM and shear wave velocity (SWV) for long curing times. Six fly ashes with different physical properties and chemical components are used for preparing the CLSM mixtures. The air content, unit weight, flowability, and setting time of CLSM are measured. Unconfined compressive strength (UCS) and elastic modulus (E) are obtained from unconfined compressive tests, and SWV (V_s_) is determined using a bender element-based wave measurement system. Experimental results show that the stiffness and strength characteristics of CLSM are relevant to the contents of two oxides (SiO_2_ and Al_2_O_3_) and the fineness of fly ash. Because the evolution of SWV is influenced by the fly ash type, the relationships UCS-V_s_ and E-V_s_ are well established. Thus, considering the fly ash type, shear wave monitoring may be effectively used for estimating strength and stiffness characteristics of CLSM.

## 1. Introduction

Controlled low-strength material (CLSM) is known as a self-compacting, flowable, and cementitious material used mainly for trench backfill, void fill, structural fill, and pavement bases. Flowability is a key property of CLSM used to determine its efficiency during construction. As an alternative to soil compaction, using CLSM can be an effective strategy to reduce labor costs, accelerate the construction process, and improve access to narrow areas. The American Concrete Institute committee 229 specified that the 28-day compressive strength of CLSM should be smaller than 8.3 MPa [1]. For the excavatable CLSM, the maximum long-term compressive strength is generally required to be less than 2.1 MPa [1]. In addition, the CLSM can be produced using industrial byproducts for resource recycling.

CLSM is typically mixed with low cement content and a high volume of fine aggregates and water. For high flowability and low strength, CLSM generally includes a high volume of fly ash in the mixture because using fly ash can improve the flowability of CLSM [2]. Fly ash, which has a spherical particle shape, lower specific gravity, and lower pozzolanic reactivity, functions as a binder or fine aggregate in a CLSM mixture [3,4,5,6]. Compared with plain Portland cement, mixing Portland cement with fly ash retards the second peak on calorimetric curves and reduces the intensity of heat dissipation rates [7,8,9]. Further, the increased percentages of fly ash and the increased water–cement ratio cause the increment in setting times of CLMS mixtures [8]. Gabr and Bowders [10] showed the physical and strength properties of CLSM prepared with Class F fly ash and sludge. According to previous studies, Class F and high carbon fly ashes demand more water and ash content for a same flowability under a given cement content [3,4]. However, fly ash from each thermal power plant may have different physical and chemical characteristics, which can influence the physical and strength properties of CLSM.

Various testing methods for evaluating the engineering properties of CLSM have been studied [11,12,13,14,15]. Han et al. [11] investigated the relationship between the unconfined compressive strength (UCS) of CLSM and compressional wave velocity estimated from using ultrasonic transducers. To overcome the limits of the ultrasonic wave velocity method based on the compressional wave, embedded piezoelectric transducers were used [12,13]. Han et al. [12] showed the evolution of shear wave velocity (SWV) along the curing time for the CLSMs mixed with the sand and silt at three different ratios. Byun et al. [13] focused on the characterization of the early age properties of CLSM based on piezo disk element and bender element and reported the consideration on the geometric boundary condition of the container in wave characterization. The hydration process of CLSM was also monitored using time-domain reflectometry, and the relationship between the dielectric constant and UCS was suggested [14]. Furthermore, direct shear tests of CLSM and soils were performed to evaluate the interface friction between CLSM and natural soils that could be around the trenches filled with CLSM in the field [15]. However, the relationships between the mechanical properties of CLSM and SWV for long curing time have not been investigated yet. The effect of fly ash type on the engineering properties of CLSM also remains poorly understood.

This study aims to investigate the effect of fly ash type on the strength and stiffness characteristics of CLSM and to present the relationships between SWV and the mechanical properties of CLSMs mixed with different fly ashes. First, fly ash type and mix design of CLSM are introduced, followed by the material properties of CLSM, such as the air content, unit weight, flowability, and setting time. Then, the test setup and results of the unconfined compressive tests (UCTs) and shear wave monitoring are explained. Finally, the strength and stiffness characteristics of CLSM are compared with the SWV.

## 2. Materials

### 2.1. Fly Ash

**Figure 1:**
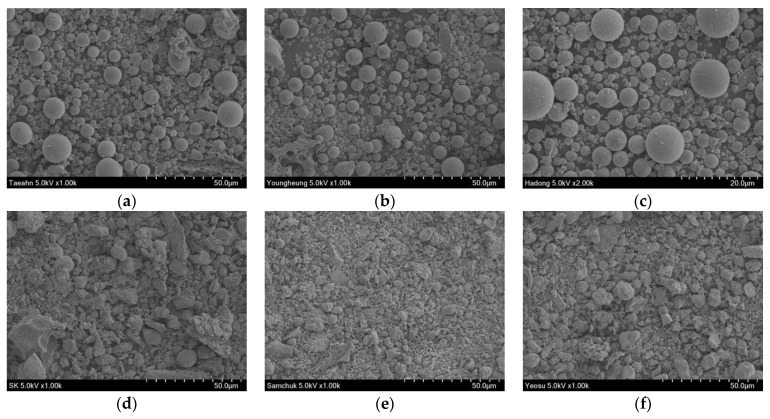
Scanning electron microscope images of fly ashes used in this study—(**a**) F1; (**b**) F2; (**c**) F3; (**d**) F4; (**e**) F5; (**f**) F6.

Fly ash is one of the materials used for the CLSM mixture. In this study, six different fly ashes (F1–F6) generated from thermal power plants with various fuel types, such as anthracite and sub-bituminous coals, were selected to investigate the effect of fly ash characteristics on developing the strength and stiffness of CLSM. The physical properties and chemical components of fly ashes are summarized in Table 1. The fly ashes mainly contain SiO_2_, Al_2_O_3_, Fe_2_O_3_, and CaO with a small amount of MgO, SO_3_, K_2_O, V_2_O_5_. According to the standards specified in ASTM C618 [16], F1–F3 can be classified as Class F fly ash. For the three fly ashes (F1–F3), the summation of SiO_2_, Al_2_O_3_, and Fe_2_O_3_ is greater than 70%. Among the three fly ashes, the fineness of F1 was the highest, whereas that of F3 was the lowest. F4 and F5 were classified as Class C fly ash. F5 was the finest among the six fly ashes. For F6, the total amount of SiO_2_, Al_2_O_3_, and Fe_2_O_3_ was lower than 50%, and the calcium content was higher than 40%. Thus, F6 was unable to be classified based on the standards of ASTM C618 [16]. Figure 1 shows the particle shape of the six fly ashes in the scanning electron microscopy (SEM) images. Most particles in F1, F2, and F3 show spherical shapes, whereas those of the particles in F4, F5, and F6 present non-spherical shapes.

### 2.2. Mix Proportion

**Figure 2:**
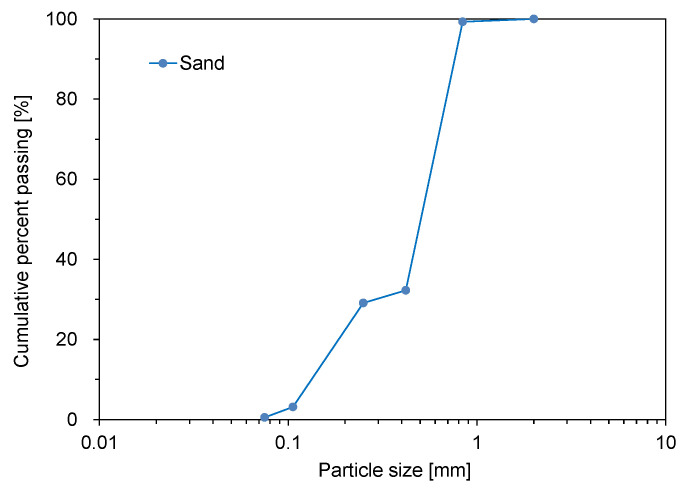
Particle size distribution of sand.

The CLSM used in this study consists of cement, fly ash, sand, calcium sulfoaluminate (CSA) expansive agent, accelerator, and water. Figure 2 shows the particle size distribution of sand, which was classified as SP according to the unified soil classification system [17]. A gap-graded particle size distribution was obtained because two different sizes of sands were mixed. For the sand, the median diameter (D_50_), coefficient of curvature (C_c_), and coefficient of uniformity (C_u_) were 0.53, 1.0, and 4.1 mm, respectively. Ordinary type I Portland cement, CSA expansive agent, and fly ash were used as binders. The CSA expansive agent mainly consists of 3CaO·3Al_2_O_3_·CaSO_4_, CaO, and CaSO_4_. An alkali-free accelerator was also used to reduce the setting time of CLSM. The mixing ratio of CLSM selected in this study is described in Table 2. For the specimen preparation, fly ash, sand, cement, and expansive agent in dry conditions were mixed, and then water and accelerator were added. The whole mixing process was finalized within 3 min. The CLSM mixtures with the six fly ashes were named M1–M6, respectively.

### 2.3. Air Content and Unit Weight

The air contents of the CLSM mixtures with the six fly ashes were measured based on the testing method specified in ASTM D6023 [18]. The values of air content ranged from 0.4% to 1.8% (Table 3). Overall, the values for air content of CLSM were in the range of the air content for a typical CLSM proposed by Folliard et al. [19]. For the CLSM mixtures with Class F fly ashes (M1–M3), the air content increased with a decrease in the loss of ignition (LOI), which matched well with the results reported by Moon et al. [20].

The unit weight of CLSM was evaluated according to ASTM D6023 [18], and the unit weight values of the specimens (M1 to M6) are summarized in Table 3. The six specimens showed the unit weights of 1.51 to 1.60 t/m^3^, which satisfied the minimum values suggested by the ACI committee 229-R99 [1]. According to Moon et al. [20], the unit weight of mortar-containing fly ash was primarily influenced by the amount of fly ash. Notably, the amount of fly ash for the CLSM mixtures used in this study was maintained.

### 2.4. Flowability

The flow test was conducted twice for each specimen according to ASTM D6103/D6103M [21]. The flowabilities of the CLSM mixtures are summarized in Table 4. The flowabilities of the CLSM mixtures ranging from 228 to 288 mm were greater than the minimum value for high flowability suggested by the ACI committee 229-R99 [1]. Both M2 and M3 showed the highest flowabilities among the six specimens, whereas M5 presented the lowest flowability. Considering the SEM images of fly ashes, the spherical particle shapes of F2 and F3 improved the flowability of the CLSM mixture more than the non-spherical particle shapes of F5. In addition to the particle shape, the flowability of CLSM was affected by the fineness and LOI of fly ash. For M5 and M6 with the two highest LOIs, their flowabilities were lower than those of other mixtures, which agreed well with the previous results reported by Kim et al. [22]. Jamkar et al. [23] also showed that the flowability increased with fly ash fineness. Although M5 and M6 had the highest fineness, their lower flowabilities were more affected by LOI than the fineness of fly ash.

### 2.5. Setting Time

**Figure 3:**
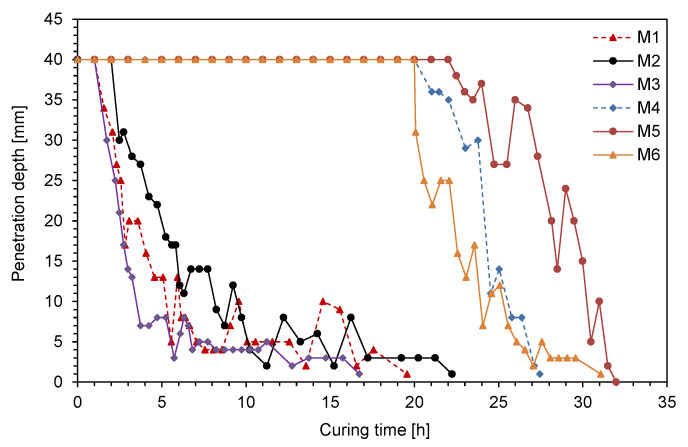
Variation in penetration of the needle along the curing time.

The setting time was estimated by performing the Vicat needle test. According to ASTM C191 [24], the needle tests were conducted every 15 min after curing, and the needle penetration was located 6-mm away from the previous testing positions of specimens. Figure 3 shows the penetration depth along the curing time. As the curing time elapsed, the penetration depths for M1–M3 started to decrease earlier, compared with those for M4–M6. The decreasing rate of penetration depth depended on the fly ash type. Notably, M1–M3 contained Class F fly ashes, whereas M4–M6 included high-calcium fly ashes. The initial and final setting times of the mixtures are summarized in Table 5. The initial setting times for M1–M3 were shorter than those for M4–M6. However, the difference in the final setting times between Class F and high-calcium fly ashes was smaller than that in initial setting times between the mixtures. Thus, the difference between the initial and final setting times for M1–M3 were longer than those for M4–M6. Considering that only the type of fly ash was changed in this study, the setting times in CLSM should be affected by the mechanical or chemical properties of fly ash. The early setting time of M1–M3 can be attributed to higher contents of Al_2_O_3_, because the aluminates hydrate at a much faster rate than silicates [25].

## 3. Experimental Study

### 3.1. Unconfined Compressive Test

For the UCT, cylindrical specimens with diameter and height of 50 and 100 mm, respectively, were used after curing for 1, 3, 7, 14, 28 and 91 days. The UCT was conducted at the loading rate of 1 mm/min using a universal testing machine with a maximum capacity of 300 kN (Kyungsung Testing Machine, KSU-30M). A servo motor in the universal testing machine was used to precisely control the load and displacement. The displacement of the specimen was measured by a rotary encoder.

**Figure 4:**
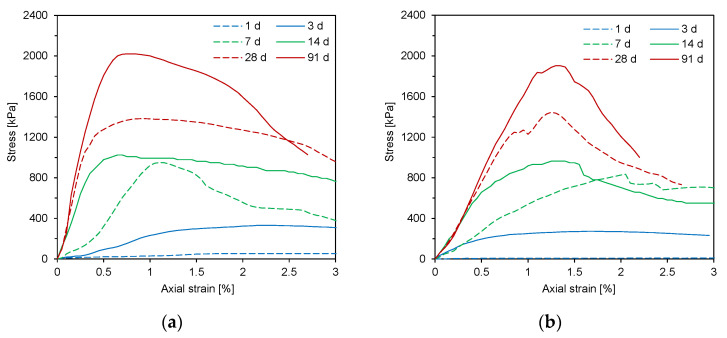
Typical stress–strain curves along the curing time—(**a**) M1; (**b**) M5.

**Figure 5:**
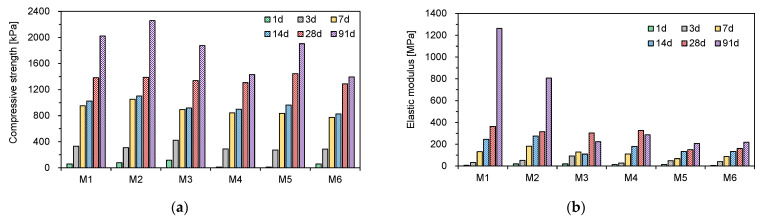
Results of unconfined compressive strength tests along curing time in different CLSM mixtures—(**a**) unconfined compressive strength; (**b**) elastic modulus.

The stress–strain curves obtained at different curing times are plotted in Figure 4. Overall, the axial stress increased to the peak value on the stress–strain curve. As the curing time increased, the initial slope before the peak value of the axial stress increased, depending on the fly ash type. The mixtures cured for 1 and 3 days showed ductile behavior without peak stress, whereas those cured for 7–91 days represented brittle behavior. After correcting the stress–strain curves for the concave upward shape, the UCS and elastic modulus for the CLSM mixtures were estimated according to the curing time (Figure 5). Figure 5a shows that the UCS increased with the curing time. The increasing rate of UCS depended on both curing time and fly ash type. The increasing rate of UCS between 1 and 7 days was greater than that of UCS between 7 and 14 days. Compared to the UCS at 14 days, the increment of UCS at 28 and 91 days was significant. The values of UCS for all specimens satisfied the requirement for the long-term compressive strengths of CLSM suggested by the ACI committee 229 [1]. At 28 days, the mixture M5 showed the greatest UCS among the specimens. According to the results of previous studies [22,26], high fineness leads to high UCS. Of note is that M5 includes the finest fly ash (F5). At 91 days, M1 and M2 showed the highest UCS. The high contents of SiO_2_ in F1 and F2 may lead to the higher strength development, because the silicates are known to play a dominant role in determining the hardening characteristics [25]. In this study, the effect of higher contents of SiO_2_ for the Class F fly ashes (M1–M2) was significant on the long-term compressive strengths of CLSM more than that of higher contents of CaO for the Class C fly ashes (M4–M6).

Cementitious materials generally show the nonlinear elastic behavior in the stress–strain curve, and the stiffness characteristic of cementitious material can be represented with secant modulus. In this study, the secant modulus was selected as the elastic modulus of CLSM [27]. The secant modulus can be determined from half of the UCS divided by the strain corresponding to the half strength. Figure 5b shows the elastic modulus of the six mixtures along the curing time. Generally, the elastic modulus increased with the curing time. The increase in the elastic modulus with the curing time depended on the fly ash type. At 91 days, M1 and M2 showed the highest elastic modulus. Considering that F1 and F2 showed the highest value of the sum of SiO_2_ and Al_2_O_3_, the two oxides might be related to long-term CLSM stiffness. M5 and M6 with the two finest fly ashes (F5 and F6) showed the two lowest elastic moduli. From the results, it was found that the SiO_2_ and Al_2_O_3_ contents and the fineness of fly ash could influence the stiffness and strength of CLSM.

### 3.2. Shear Wave Characterization

**Figure 6:**
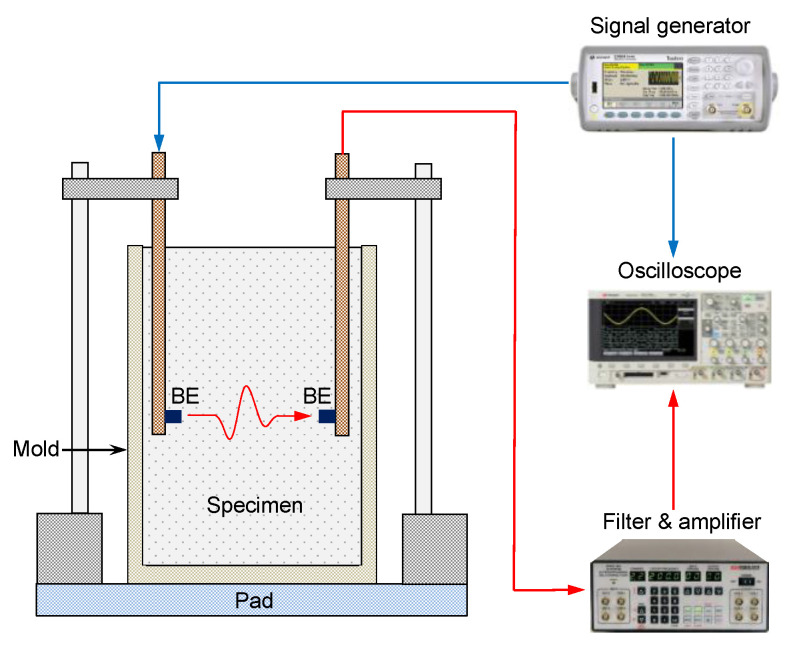
Schematic drawing of test setup for the shear wave measurement.

In this study, a pair of bender elements were used to monitor the characteristics of the shear waves in the CLSM mixture along the curing time. The parallel type of bender element was selected for reducing the electrical interference. To minimize electrical interference, conductive paint and grounding were also applied to the bender elements. Figure 6 shows a wave measurement system and a CLSM specimen prepared in a polyvinyl chloride mold with a diameter, height, and thickness of 60, 120, and 4.5 mm, respectively. In the PVC mold, a pair of bender elements were installed in rectangular copper pipes, which were fixed with epoxy and silicone to behave as a cantilever beam. A rubber pad was placed under the PVC mold to prevent any interference with the waves reflected from the bottom of a testing plane during the wave measurement. The measurement system comprised a signal generator, a filter-amplifier, and an oscilloscope. A step signal with an amplitude of 10 V and a frequency of 20 Hz was set as the input through the signal generator, and a shear wave was propagated through a specimen of CLSM. The received signal at a bender element on the other side was filtered to remove noise with frequencies lower than 500 Hz and higher than 100 kHz through the filter-amplifier, and the output signal was finally recorded on the oscilloscope.

**Figure 7:**
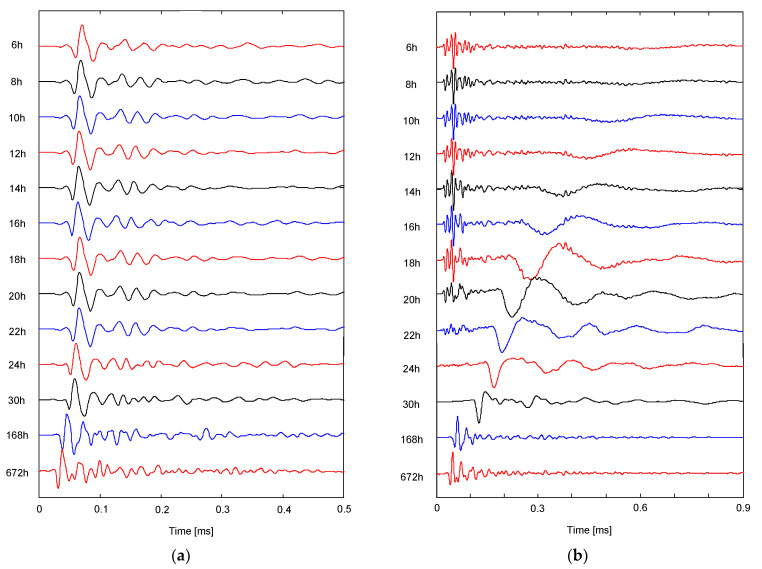
Typical signals obtained from the specimens of (**a**) M3 and (**b**) M6.

The output signals obtained at two CLSM mixtures along the curing time from 6 h to 28 days are plotted in Figure 7. To investigate the change in the shear wave, the output signals were normalized with their maximum amplitudes at each curing time. For M3, the shear wave was significant on the output signals, even at an early curing time, because the initial setting time was 2.2 h. Notably, the shear wave is propagated only through the solid, whereas the compressional wave is propagated through both solid and fluid. The first arrival time of the shear wave for M3 slightly decreased as the curing time increased. By contrast, for M6, the shear waves at curing times of 6–10 h rarely occurred, and instead, the compressional waves were detected only on the output signals. As the curing time increased, the shear waves were gradually significant on the signals, and the first arrival times of shear waves rapidly decreased compared with those for M3. Since the output signals were normalized with their maximum amplitudes and the amplitude of compressional wave at the curing times after 1 day was significantly smaller than that of the shear wave, the compressional waves could not be revealed on the signals.

**Figure 8:**
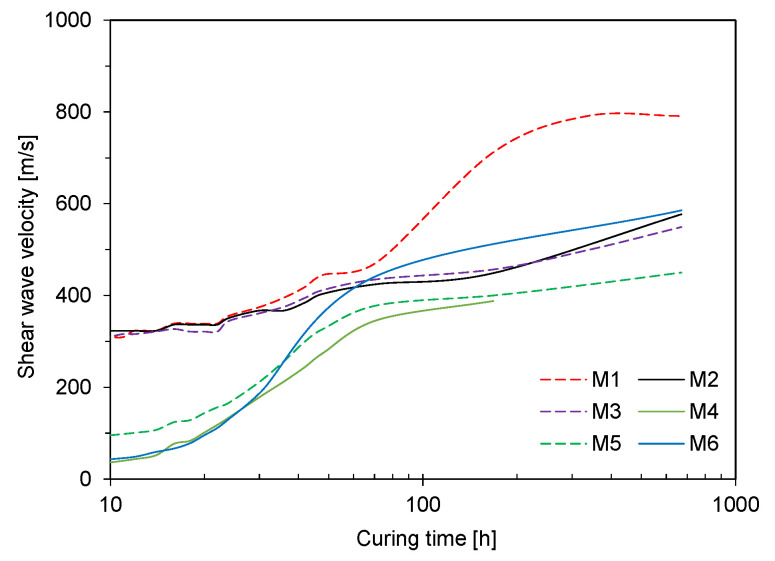
Variation in shear wave velocity along the curing time.

SWV is determined by dividing the travel distance of shear wave by its first arrival time. In this study, tip-to-tip distance between two bender elements was selected as the travel distance. The SWV along the curing time is plotted in Figure 8. At the curing time of 10 h, SWVs for M1–M3 were greater than those for M4–M6. After 20 h, SWVs for M4–M6 rapidly increased with the curing time. Notably, the initial setting times for M4–M6 ranged from 20.6 to 27.6 h, and the differences between the initial and final setting times for M4–M6 were shorter than those for M1–M3. After the curing time of 3 days, the increasing rates of SWVs for M2–M6 were similar. For M1, the SWV significantly increased after 3-days curing, which concurred with the findings on the elastic modulus obtained from UCTs.

### 3.3. Comparison

**Figure 9:**
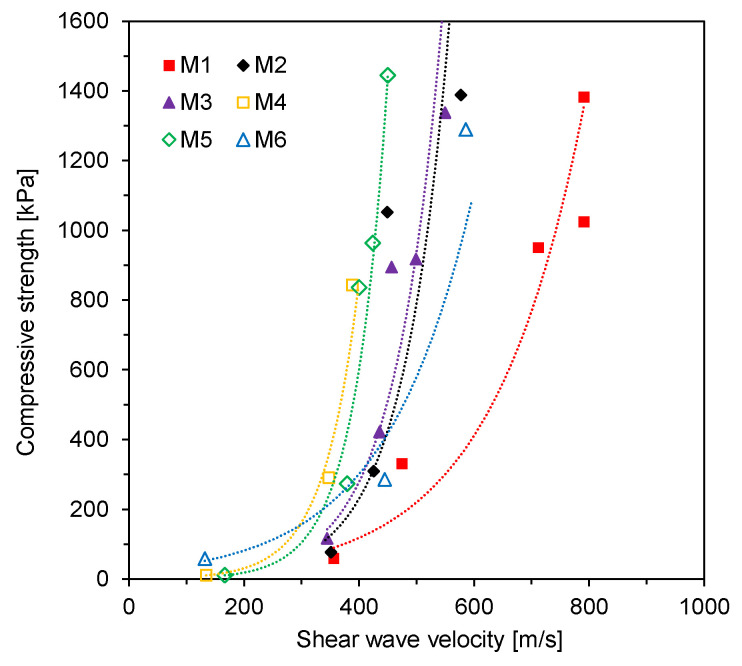
Compressive strength versus shear wave velocity.

For the six mixtures, both UCS measured from UCT and SWV estimated from shear wave monitoring are plotted in Figure 9. Previous studies have used the exponential function relationships between the UCS or elastic modulus and elastic wave velocity in cementitious materials [28,29,30,31]. Lee et al. [31] showed that the relationships between the UCS or elastic modulus and shear wave velocity followed the exponential function. Thus, in this study, the relations between UCS and SWV for each mixture were established based on exponential function as follows:

(1) $$\mathrm{UCS}=\;\alpha e^{\beta V_{s}}$$

where α and β are the parameters of the exponential function relationship. The values of α and β determined by regression analysis for the six mixtures are summarized in Table 6. The values of α and β depended on the fly ash type in the CLSM mixture. For each mixture, the exponential function relationship between UCS and SWV was well established with the coefficients of determination of greater than 0.76, and the equation can be used to estimate the UCS of CLSM at a curing time from measuring shear waves.

**Figure 10:**
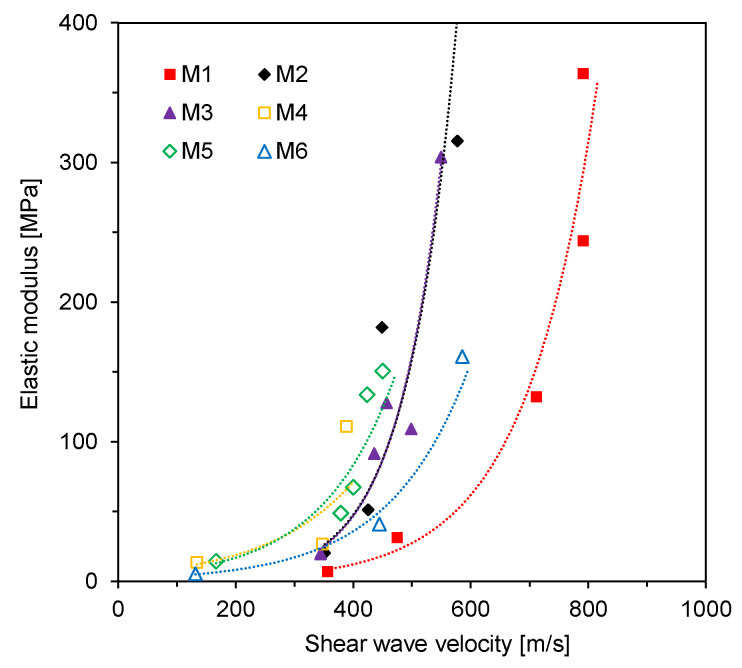
Elastic modulus versus shear wave velocity.

The elastic moduli estimated from the UCT and the SWV are plotted in Figure 10. The relations between the elastic modulus and SWV were established with an exponential function as follows:

(2) $$E=\;\gamma e^{\delta V_{s}}$$

where γ and *δ* are the parameters of the exponential function relationship. The values of γ for M1–M3 with Class F fly ashes were smaller than those for M4–M6 with high-calcium fly ashes, whereas the values of *δ* for M1–M3 were greater than those for M4–M6. The results mean that, especially for higher stiffness, the elastic modulus characterization based on shear waves in the CLSM with Class F fly ashes is less sensitive than that in the CLSM with high-calcium fly ashes. Notably, as the exponent increased, the trends became steeper away from the origin.

## 4. Summary and Conclusions

This paper explored the strength and stiffness characterization of fly ash-based CLSM using shear waves. Six fly ashes with different physical properties and chemical components were used to investigate the effect of fly ash characteristics on the development of strength and stiffness of the CLSM. The first three fly ashes with spherical particle shapes were classified as Class F, whereas the other three fly ashes had non-spherical particle shape and high calcium content. The flowability of CLSM was primarily influenced by the particle shape and LOI of fly ashes. The setting times for Class F fly ashes were longer than those for high-calcium fly ashes, which is attributable to the content of chemical components of fly ashes. For the mechanical properties of CLSM, the increasing rate of UCS depended on fly ash type and curing time. The stiffness and strength characteristics of CLSM were relevant to the contents of the two oxides (SiO_2_ and Al_2_O_3_) and the fineness of fly ash. Shear wave monitoring system including a pair of bender elements was used for the characterization of CLSM. Initially, SWVs for the CLSMs with Class F fly ashes were greater than those for the CLSMs with high-calcium fly ashes. After a 3-day curing, the increasing rates of SWVs for five CLSM mixtures were similar, except for M1. Based on the exponential function, the relationships between UCS and SWV were well established. The exponential function relationships between elastic modulus and SWV were also proposed to estimate the stiffness characteristics of CLSM along the curing time. In particular, the values of the coefficient and exponent of the relationships were relevant to the fly ash type. Therefore, the shear wave monitoring may be effectively used for estimating strength and stiffness characteristics of CLSM, considering the fly ash type.

## Figures and Tables

**Table 1:** Physical properties and chemical components of fly ashes.

Fly Ash	Physical Property		Chemical Component (%)								
	Density (g/cm^3^)	Fineness (cm^2^/g)	SiO_2_	Al_2_O_3_	Fe_2_O_3_	CaO	SO_3_	MgO	K_2_O	V_2_O_5_	LOI
F1	2.3	3790	66.52	17.82	6.33	3.47	0.00	0.00	0.47	3.27	3.27
F2	2.3	3600	68.90	17.67	3.08	4.27	0.00	0.00	0.80	3.17	3.17
F3	2.3	3320	60.70	17.61	10.61	5.67	0.00	0.53	0.63	2.15	2.15
F4	2.4	3610	48.48	8.13	2.53	30.93	4.86	0.00	0.68	2.28	2.28
F5	2.9	4860	34.81	9.00	11.30	27.81	4.77	5.82	0.13	4.26	4.26
F6	2.3	4260	25.79	2.83	13.72	40.03	4.65	7.46	0.00	3.40	3.40

**Table 2:** Mix design for CLSM used in this study.

Component	Water	Cement	Sand	Fly Ash	CSA	Accelerator
Proportion (kg/m^3^)	596.7	171.8	716	83.5	43	35.8

**Table 3:** Air contents and unit weights of the CLSM mixtures with six different fly ashes.

CLSM Mixture	M1	M2	M3	M4	M5	M6
Air content (%)	1.8	1.3	0.2	0.8	0.4	0.9
Unit weight (kN/m^3^)	15.7	15.5	15.1	15.6	15.0	14.8

**Table 4:** Flowability of the CLSM mixtures with six different fly ashes.

CLSM Mixture	M1	M2	M3	M4	M5	M6
Flowability (mm)	260	288	288	280	228	250

**Table 5:** Setting times of the CLSM mixtures.

Setting Time (h)	M1	M2	M3	M4	M5	M6
Initial	2.6	4.0	2.2	24.0	27.6	20.6
Final	19.6	22.2	16.7	27.5	32.0	31.1

**Table 6:** Parameters of relationships between mechanical properties and shear wave velocity of CLSM.

Mixture	Compressive Strength			Elastic Modulus		
	α	β	R^2^	γ	δ	R^2^
M1	9.6981	0.0062	0.9107	0.4673	0.0081	0.9755
M2	1.6722	0.0123	0.7641	0.3772	0.0121	0.8374
M3	2.1661	0.0121	0.9127	0.3207	0.0124	0.9193
M4	1.1124	0.0166	0.9925	4.9539	0.0067	0.7102
M5	0.5569	0.0175	0.9817	3.3924	0.0080	0.9142
M6	22.049	0.0065	0.9609	1.8649	0.0074	0.9899
